# Salicylic Acid in Foul Breath

**Published:** 1876-04

**Authors:** 


Salicylic Acid for Foul Breath and Offensive Expectoration—*Dr. Da Costa.*—This patient is brought before you to show you that there is destruction of pulmonary structure, and to call your attention to the fact that the peculiar bad odor belonging to the breath and the expectoration where there is breaking down of the lung or pulmonary abcess, is absent in this case now, although it was previously a very noticeable symptom. Being struck with the foulness of the patient's breath, which was so offensive as to discourage physical examination of the chest, I determined to try salicylic acid. The dose given was five grains, dissolved in half an ounce of water, with the aid of a drachm of glycerine. This was exhibited three times a day, with the effect of purifying the expiration, and improving the character of the expectoration, although upon the latter the influence has not been quite so well marked.

If in salicylic acid we have an agreeable and harmless remedy against foul breath, and one that will also exercise a detergent effect on the character of offensive secretions, you will see at once that a long felt want is supplied. In gangrene of the lung, for instance, the characteristic breath poisons the patient and renders his presence insupportable to those around him; if salicylic acid will change this, as it has done in the case before us, a not unimportant application of this comparatively new agent is presented. If internal administration does not accomplish the desired result, it can be used in the atomizer in the solution before mentioned (grains x to water 1 oz., with sufficient glycerine to make it soluble.) About eighteen months ago I first used it as a deodorizer, in a woman with stomach disorder accompanied by very offensive breath, and with the happiest results. In cases of indigestion, with bad breath, I have used it a number of times since, with great success. This is a legitimate application of this disinfectant; and other similar conditions will suggest themselves to you, as in fetid bronchitis, in abscess, or in cancer of the lung with offensive discharges, where it will prove of great value. My experience with the agent, on the whole, is that in these complaints it modifies the breath greatly, and changes the offensive character of the expectoration, though to a less degree than that of the breath. It has more influence on the sputum in fetid bronchitis, than it has in diseases attended with destruction of the lung.—*Med. Brief.*

